# The challenges facing indigenous communities in Latin America as they confront the COVID-19 pandemic

**DOI:** 10.1186/s12939-020-01178-4

**Published:** 2020-05-07

**Authors:** Sergio Meneses-Navarro, María Graciela Freyermuth-Enciso, Blanca Estela Pelcastre-Villafuerte, Roberto Campos-Navarro, David Mariano Meléndez-Navarro, Liliana Gómez-Flores-Ramos

**Affiliations:** 1 CONACYT/Center for Health Systems Research, Center for Population Health Research, National Institute of Public Health, Universidad No. 655, C.P, 62100 Cuernavaca, Morelos Mexico; 2 Center for Research and Advanced Studies in Social Anthropology, Southeast Campus, Carretera a San Juan Chamula kilómetro 3.5, Barrio La Quinta San Martín, CP. 29247 San Cristóbal de Las Casas, Chiapas Mexico; 3 Center for Health Systems Research, National Institute of Public Health, Universidad No. 655, C.P. 62100 Cuernavaca, Morelos Mexico; 4 National Autonomous University of Mexico, Circuito Escolar 411A, Copilco Universidad, Coyoacán, 04360 Ciudad de México, Mexico; 5 Committee for the Promotion of Safe Motherhood in Mexico, Carretera a San Juan Chamula kilómetro 3.5, Barrio La Quinta San Martín, CP. 29247 San Cristóbal de Las Casas, Chiapas Mexico; 6 CONACYT/Center for Population Health Research, National Institute of Public Health, Universidad No. 655, C.P. 62100 Cuernavaca, Morelos Mexico

**Keywords:** COVID-19, Indigenous populations, Latin America, Health systems response

## Abstract

The coronavirus disease 2019 (COVID-2019) pandemic struck Latin America in late February and is now beginning to spread across the rural indigenous communities in the region, home to 42 million people. Eighty percent of this highly marginalized population is concentrated in Bolivia, Guatemala, Mexico and Peru. Health care services for these ethnic groups face distinct challenges in view of their high levels of marginalization and cultural differences from the majority. Drawing on 30 years of work on the responses of health systems in the indigenous communities of Latin America, our group of researchers believes that countries in the region must be prepared to combat the epidemic in indigenous settings marked by deprivation and social disparity. We discuss four main challenges that need to be addressed by governments to guarantee the health and lives of those at the bottom of the social structure: the indigenous peoples in the region. More than an analysis, our work provides a practical guide for designing and implementing a response to COVID-19 in indigenous communities.

## Resumen

La pandemia de coronavirus 2019 (COVID-19) golpeó a América Latina a fines de febrero y ahora está comenzando a extenderse por las comunidades indígenas y rurales de la región, hogar de 42 millones de personas. El 80% de esta población altamente marginada se concentra en Bolivia, Guatemala, México y Perú. Los servicios de atención médica para estos grupos étnicos enfrentan desafíos distintos en vista de sus altos niveles de marginación y diferencias culturales de la mayoría. Con más de 30 años de trabajo e investigación sobre las respuestas de los sistemas de salud dirigida a las comunidades indígenas de América Latina, consideramos que los países de la región deben estar preparados para combatir la epidemia en contextos indígenas marcados por la privación y la disparidad social. Discutimos cuatro desafíos principales que deben ser abordados por los gobiernos para garantizar la salud y la vida de los que se encuentran en la parte inferior de la estructura social: los pueblos indígenas de la región. Este análisis proporciona una guía práctica para diseñar e implementar una respuesta a COVID-19 en comunidades indígenas.

## Main text

Originating in Wuhan, China, last December, the coronavirus disease 2019 (COVID-2019) pandemic struck Latin America in late February and is now spreading to the rural indigenous communities in the region, home to 42 million individuals. Eighty percent of this highly marginalized population is concentrated in Bolivia, Guatemala, Mexico and Peru. Rural indigenous peoples have historically encountered the steepest barriers to health services and endured profound discrimination based on ethnicity, poverty and language. Severe cases of COVID-19 are expected to sweep through these communities given their precarious health and living conditions. Malnutrition, infectious diseases and chronic illnesses characterize their epidemiological profile and are compounded by high fertility levels. The attack rate of the epidemic in Latin America has been estimated at 0.2%. Of the 84,000 expected patients, approximately 60,000 will seek health care, 8400 will require hospitalization and 3600 will be classified as critical. Governments have focused their responses primarily on urban populations speaking the dominant languages: Spanish and Portuguese. However, they have shown little interest in crafting strategies targeted specifically for the rural and indigenous communities.

Drawing on 30 years of work on the responses of health systems in the indigenous communities of Latin America, our group of researchers believes that countries in the region must be prepared to combat the epidemic in indigenous settings marked by deprivation and social disparity. To do so, four main challenges need to be addressed:

First, mistrust of authority and disbelief in the existence and gravity of the pandemic will likely result in the repudiation of health personnel tasked with containing the epidemic.

Second, disinformation and beliefs lacking empirical support abound during crises. Rumors and false information on the origins of the COVID-19 infection will undermine the preventive and therapeutic measures required to combat the epidemic.

Third, limited access to water will hinder compliance with even basic preventive measures such as frequent hand washing. Deficient communication obstructs access to geographically distant health-care facilities in urban centers that offer quality and timely response capacity. This acquires particular relevance in cases of severe infection. A lack of financial protection and health responsiveness with an intercultural perspective jeopardizes the effectiveness of treatment.

Finally, as a consequence of the widespread unemployment generated by the pandemic, indigenous people are migrating in large numbers from major cities and tourist sites back to their communities of origin. Many are returning from the United States, one of the global epicenters of COVID-19 infection, as well as from Europe, especially Spain. This poses a high risk of contagion for rural indigenous communities in Latin America.

To address these challenges, governments must:

First, design clearly understandable communication strategies in indigenous languages concerning the imminent extension of the pandemic to all communities in Latin America. The likelihood of severe cases should also be emphasized. This information must be grounded in scientific evidence, within a framework of respect for the indigenous worldview, while avoiding explanations of the pandemic and forms of care based on magical-religious thinking.

Second, prepare specific action plans that ensure access to diagnostic services and hospital care, where necessary. This should include communication strategies for identifying cases of suspected contagion. Plans should also provide for ground and air transportation in severe cases, free hospital services and access to medication and medical equipment (including intensive therapy and assisted breathing instruments).

Eliminating all forms of mistreatment, such as discrimination by reason of race or social class, should constitute the cross-cutting axis of all responses formulated by health systems throughout Latin America to halt the spread of the virus in rural communities. The COVID-19 pandemic is crippling normal life around the world. Even in the face of the resulting uncertainty, Latin American countries have a responsibility, rooted in history, to guarantee the health and lives of those at the bottom of the social structure, including the indigenous peoples in the region.

## Data Availability

Not applicable.

